# ZnO-nanorods/graphene heterostructure: a direct electron transfer glucose biosensor

**DOI:** 10.1038/srep32327

**Published:** 2016-08-30

**Authors:** Yu Zhao, Wenbo Li, Lijia Pan, Dongyuan Zhai, Yu Wang, Lanlan Li, Wen Cheng, Wei Yin, Xinran Wang, Jian-Bin Xu, Yi Shi

**Affiliations:** 1 School of Electronic Science and Engineering, Collaborative Innovation Center of Advanced Microstructures, Nanjing University, Nanjing 210093, China; 2 School of Chemistry and Chemical Engineering, Nanjing University, Nanjing 210093, China; 3 Department of Electronic Engineering, The Chinese University of Hong Kong, Shatin, New Territories, Hong Kong SAR, China

## Abstract

ZnO-nanorods/graphene heterostructure was synthesized by hydrothermal growth of ZnO nanorods on chemically reduced graphene (CRG) film. The hybrid structure was demonstrated as a biosensor, where direct electron transfer between glucose oxidase (GOD) and electrode was observed. The charge transfer was attributed to the ZnO nanorod wiring between the redox center of GOD and electrode, and the ZnO/graphene heterostructure facilitated the transport of electrons on the hybride electrode. The glucose sensor based on the GOD-ZnO/CRG/Pt electrode had a high sensitivity of 17.64 μA mM^−1^, which is higher than most of the previously reported values for direct electron transfer based glucose biosensors. Moreover, this biosensor is linearly proportional to the concentration of glucose in the range of 0.2–1.6 mM. The study revealed that the band structure of electrode could affect the detection of direct electron transfer of GOD, which would be helpful for the design of the biosensor electrodes in the future.

In recent years, amperometric glucose biosensors have attracted intensive attention and have been extensively studied because of their important applications in healthcare, food industry, chemical, and biological analysis^1^,^2^,^3^,^4^,^5^. In an amperometric glucose biosensor, electron transfer between glucose oxidase (GOD) and electrodes can be realized by redox mediators or direct electron transfer (DET)^6^. Because redox mediators based biosensors have many critical limitations, such as high cost, potential cytotoxicity, and low selectivity^7^, direct electrochemistry biosensor is considered to be the choice of next generation of biosensor for its reversible nature and low interference resulted from its low redox potential^8^. However, it still remains a challenge to achieve direct electrochemistry of most redox enzymes on bare solid electrodes because redox centers of the enzymes are deeply buried in the proteins and the biological matrix is instable upon interaction with the electrode surface^7^,^9^. Nanostructured materials have shown great potential for direct electron transfer because some of them may wire between the redox center of enzymes and the electrodes^10^,^11^,^12^,^13^,^14^,^15^.

Graphene possesses a large specific surface area (up to 2630 m^2^ · g^−1^) to accommodate abundant biomolecule loadings, and shows the potential to impart excellent bio-detection sensitivity. The high conductivity and small bandgap features of graphene facilitate the electron transfer between the biomolecules and graphene surface^16^. On the other hand, nanostructured ZnO is also an excellent candidate for biosensor materials due to its high surface area, low toxicity, good chemical stability and biological compatibility, and high electron mobility^17^,^18^,^19^. Moreover, ZnO has a high isoelectric point (IEP) about 9.5, which makes it suitable for absorption of GOD primarily driven by electrostatic interaction^20^,^21^. Up to date, some with low IEP about 4.2 in the physiological pH, because the enzyme immobilization is primarily driven by electrostatic interaction. ZnO-nanostructure-based glucose sensors have been reported^22^,^23^,^24^,^25^,^26^, but only a few works have been reported on the direct electrochemistry of GOD immobilized on ZnO/graphene hybrid nanocomposites^27^,^28^,^29^,^30^. Moreover, to the best of our knowledge, there are no literature that is published to illustrate which kinds of metal oxides based composite materials favor the DET process from the consideration of band gap and work function of heterostructured electrodes.

In this paper, ZnO-nanorods/graphene heterostructure was prepared by growing ZnO nanorods on chemically reduced graphene (CRG) film. Direct electron transfer behavior was observed on the GOD immobilized ZnO-nanorods/graphene heterostructure, in sharp contrast to ZnO nanorods directly grown on Pt electrode and graphene/Pt electrode, for which no signal of direct electron transfer were presented. We find that not only the morphology^31^ but also the electronic band structure of functional material electrode affect the detection of direct electron transfer. Furthermore, the redox reversibility of GOD and surface controlled electrochemical process on the ZnO-nanorods/graphene heterostructure reveal its potential application for the redox-mediator-free biosensors.

## Results

**Figure 1:**
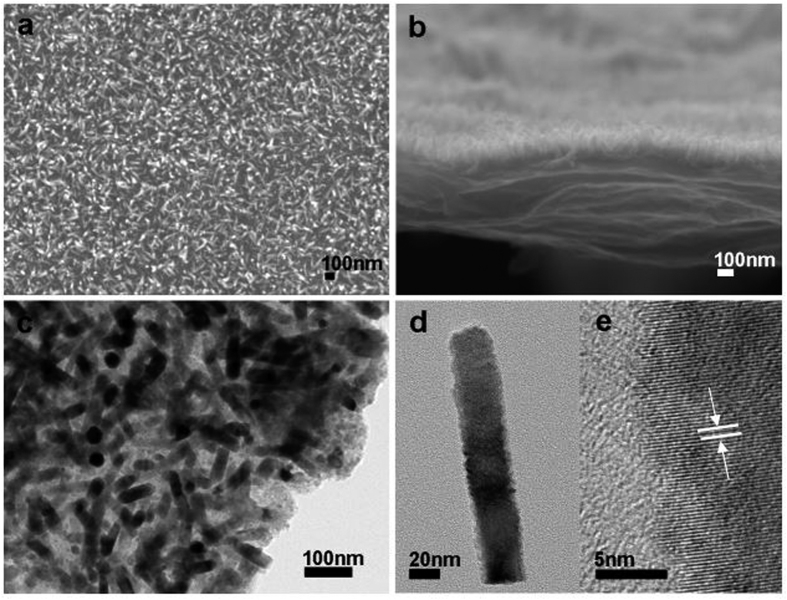
SEM and TEM images of the ZnO/graphene heterostructure. (**a**) Top view SEM image. (**b**) Cross-sectional SEM image shows the ZnO nanorods grown on graphene. (**c**) TEM image of the ZnO-nanorods/graphene heterostructure. (**d**) TEM image of a single ZnO nanorod. (**e**) HRTEM image of a single ZnO Nanorod.

Figure 1 showed typical SEM and TEM images of the ZnO-nanorods/graphene heterostructure. As shown in Fig. 1a, the top-image of SEM indicated that the ZnO nanorod arrays were produced on graphene with high uniformity and packing density, consistent with the cross-sectional SEM image as shown as Fig. 1b. The thickness of the CRG film was estimated to be about 450–600 nm. TEM image further confirmed the heterostructure of ZnO-nanorods/graphene, as shown as Fig. 1c. Figure 1d shows a single ZnO nanorod obtained by crushing the sample up by sonication. The diameter of the nanorod is about 30 nm with length about 150 nm. The HRTEM image shown in Fig. 1e indicated that the nanowire was high crystalline with a lattice spacing of 0.26 nm, which corresponded to the (002) plane in the ZnO crystal lattice (PCPDF #89-1397). Note the ZnO nanorods had a rough surface (Fig. 1d,e), which may be favorable for immobilization of GOD and wiring the proteins.

The XRD patterns of the ZnO-nanorods/graphene heterostructure and CRG film were presented in Supplementary Fig. S3. One broad reflection peak centered around 2*θ* = 25° was observed in the XRD pattern of CRG, which can be correlated to an interlayer spacing of 0.36 nm in the graphene. All diffraction peaks in the range 30° < 2*θ* < 60° for the ZnO/CRG heterostructure can be indexed to the hexagonal wurtzite structured ZnO with lattice constants *a* = 0.325 nm and *c* = 0.521 nm, which are in good agreement with the values provided by the standard JCPDS card (JCPDS 36-1451) database. As shown as Supplementary Fig. S4, the PL spectrum shows a strong peak at 382 nm for the sample of ZnO-nanorods/graphene heterostructure, which is in consistence with the band edge emission of ZnO.

**Figure 2:**
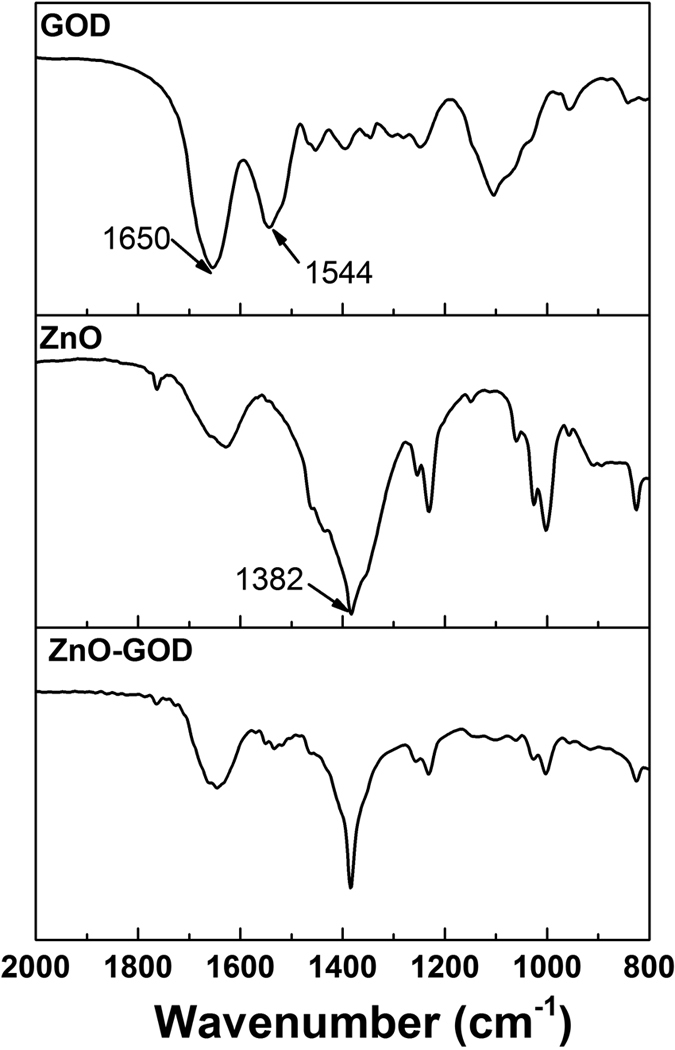
FT-IR spectra of pure GOD, ZnO nanorods, and GOD-fixed ZnO nanorods.

Fourier transform infrared spectroscopy of pure GOD, as-prepared ZnO nanorods and GOD-fixed ZnO nanorods confirmed the strong interaction between the ZnO nanorods and GOD molecules, as shown as Fig. 2 32. For pure GOD, there are some characteristic absorption bands of GOD, typical amide I absorption band round 1648 cm^−1^ and amide II absorption near 1544 cm^−1^. The ZnO nanorods exhibit obvious absorption band at 1382 cm^−1^, which attributes to the symmetrical vibration of –COO–, suggesting acetic ions are adsorbed on the surface of ZnO nanorods which came from acetic zinc used in the preparation^33^. After dropping GOD and Nafion solution, the two absorption bands of amide are appear in the IR spectrum of the ZnO nanorods, which demonstrate that GOD molecules are effectively supported on the surface of ZnO nanorods.

**Figure 3:**
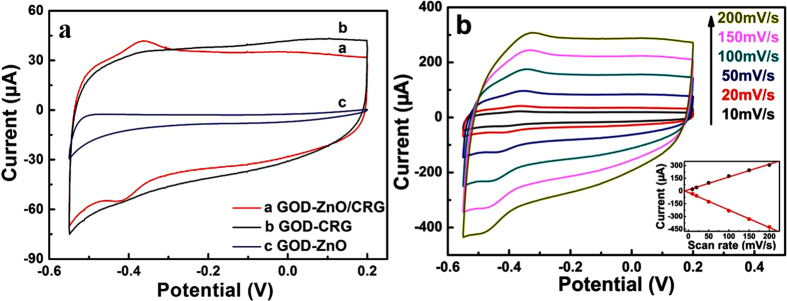
Cyclic voltammograms measurements. (**a**) Cyclic voltammograms of GOD-ZnO/CRG heterostructure, GOD-CRG and GOD-ZnO nanorods on standard Pt electrode in N_2_-saturated 0.2 M PBS solution (pH = 5.8) at a scan rate of 20 mV s^−1^. (**b**) Cyclic voltammograms of various scan rate: 10, 20, 50, 100, 150, and 200 mV s^−1^. Inset: plot of peak current vs scan rate.

The electrochemistry performances were all measured at 30 °C. Figure 3a shows cyclic voltammograms (CV) of GOD ZnO-nanorods/graphene heterostructure, and the control samples of GOD-CRG film and GOD-ZnO nanorod/Pt electrode in N_2_-saturated 0.2 M PBS solution (pH = 5.8). A pair of well-defined redox peaks was observed in the curve of ZnO/CRG (Curve a), indicating direct electron transfer between GOD and ZnO/CRG. The formal potential calculated by averaging the cathodic and anodic peak potentials was estimated to be ~−0.39 V (vs SCE), with ~61 mV peak-to-peak separation. From scan rates of 10 to 200 mV s^−1^, the cathodic peak current (*I*_pc_) and anodic peak current (*I*_pa_) increase linearly with the scan rate increase (Inset of Fig. 3b). This is attributed to the reversible surface control of GOD electrochemical reaction. According to Laviron’s equation for a surface-controlled electrochemical system (Δ*E*_p_ < 200/n mV)^23^,


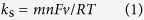


where *m* is a parameter related to the peak-to-peak separation, and is calculated to be 0.60, *n* is the transferred electrons number which is assumed to be two, *F* is the Faraday constant, *v* is the scan rate, *R* is the gas constant, and *T* is the temperature. The apparent heterogeneous electron transfer rate constant (*k*_*s*_) was estimated to be 0.92 S^−1^, suggesting the direct electron transfer of GOD had good reversibility. The effect of scan rate on the electrochemical response of the immobilized GOD is shown in Fig. 3b. The redox peak current scaled linearly to the scan rate between 10 to 200 mV s^−1^ (Fig. 3b inset). Meanwhile the cathodic and anodic peak potentials showed a small shift and Δ*E*_p_ was also gradually increased. All these characteristics suggested that the direct electron transfer between GOD and ZnO could be easily performed on the ZnO-nanorods/graphene heterostructure, and it was a surface-controlled electrochemical process^34^. Electrochemical measurements have also been performed on the controls of CRG film and ZnO nanorods/Pt, as shown as Fig. 3a (Curve b and c, respectively). No redox peaks were found in the controls, indicating that no direct electron transport was detected on the both samples.

## Discussion

The morphology of ZnO nanorods favors for the immobilization of GOD and detection of direct electronic transfer. It is well known that the active redox center of GOD is deeply embedded in a protective protein shell, which makes the direct electron transfer between GOD and electrode difficult to realize. We suggested that the nanoscale size and the accidented surface of ZnO nanorods facilitate the immobilization of GOD and wiring its redox center with the electrode. When GOD was dropped onto the surface, the protein was immobilized on ZnO by electrostatic interaction. The rough surface of the ZnO nanorods, as shown as Fig. 1d,e, should favor the absorption of GOD on ZnO nanorods and reduce the distance between the active redox center and surface of ZnO, facilitating the direct electron transfer process.

**Figure 4:**
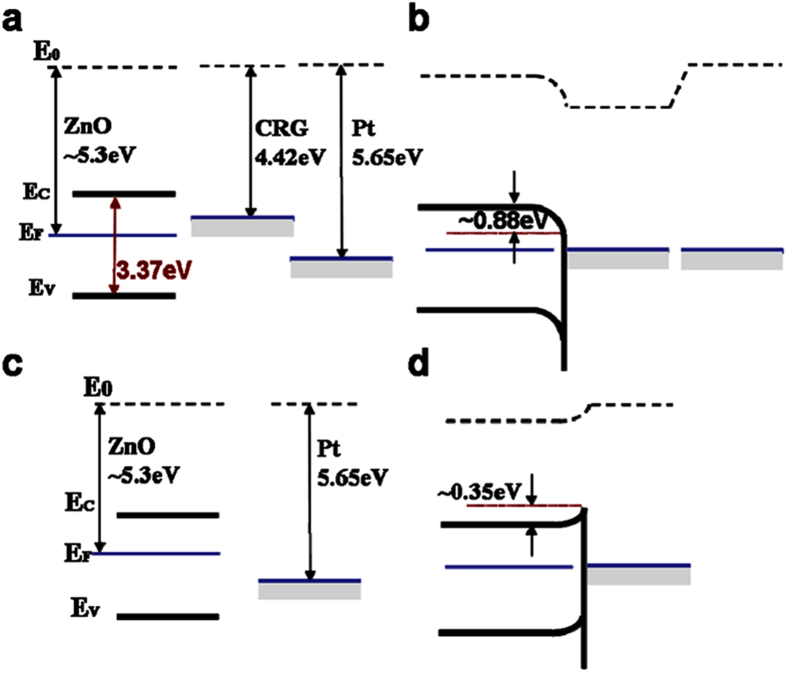
Band structure at the interface of electrodes. (**a**) Band structure before and (**b**) after physical contact of ZnO/CRG/Pt electrode, where the dipole formation at the metal-graphene interface was ignored. (**c**) Band structure at the interface before and (**d**) after physical contact of ZnO/Pt electrode.

The proper energy level alignment of ZnO/graphene heterostructure was critical to the detection of the electron transport signals. Note that no direct electrochemistry was detected for the control of ZnO-nanorods/Pt electrode, as shown in Fig. 3a. The band structures of the electrode interfaces are shown in Fig. 4. ZnO has a wide band gap about 3.37 eV, and the work function (*W*) of eigen-state ZnO, graphene and Pt are 5.3 eV^35^, 4.42 eV^36^ and 5.65 eV^37^, respectively. Figure 4a,b show the band structure before and after contact of ZnO-nanorods and graphene film. In the ZnO/graphene heterostructure, the energy band of ZnO was bent downward because the work function of ZnO was higher than that of CRG (*W*_ZnO_ > *W*_CRG_), and an electron anti-blocking layer was formed. As a result, the heterostructure would be favorable for the electron transport from GOD-ZnO to the electrode. In contrast, for the electrode structure of ZnO-nanorods grown on Pt electrode directly, the band structure before and after contact of ZnO and Pt is shown in Fig. 4c,d. The energy band of ZnO/Pt was bent upward, because the work function of ZnO is smaller than that of Pt (*W*_ZnO_ < *W*_Pt_). As a result, a Schottky barrier about 0.35 eV was formed, which blocked the electron transport from the ZnO nanorods to the Pt electrode. In addition, most of the ZnO are n-doped, its actual work function will be smaller than intrinsic ZnO, and the barrier should be larger than 0.35 eV, implying that electron would be more difficult to transfer from ZnO to Pt electrode. All of these indicate that the heterostructure of ZnO/CRG facilitates the electron transfer from ZnO to electrode, and favors for the detection of direct electrochemical signal of enzyme.

**Figure 5:**
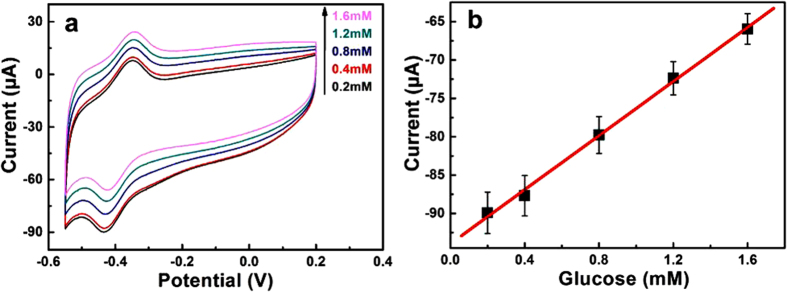
Biosensor performance. (**a**) Cyclic voltammograms of GOD-ZnO/CRG heterostructure on Pt electrode in various concentrations of glucose PBS solution saturated with O_2_: 0.2, 0.4, 0.8, 1.2, and 1.6 mM. (**b**) Calibration curve corresponding to amperometric responses at −0.43 V with a scan rate 20 mV/s.

Furthermore, we demonstrated the heterostructure of ZnO/CRG as glucose sensors. The measurement was conducted in 0.2 M PBS solution with different concentrations of glucose saturated with O_2_. Figure 5 shows the CV curves of GOD-ZnO/CRG/Pt electrode. When the concentration of glucose is gradually increased from 0.2 to 1.6 mM, the calibration curve corresponding to the amperometric response is almost linearly dependent on the concentration of glucose with a correlation coefficient (*R*) at 0.998. The sensitivity calculated from the linear portion of the calibration is 17.64 μA mM^−1^, i.e., 89.84 μA mM^−1^ cm^−2^, which is higher than most of the previously reported values for direct electron transfer based glucose biosensors^13^, and could be used for detecting the glucose content in the body fluid, such as perspiration and tears.

**Figure 6:**
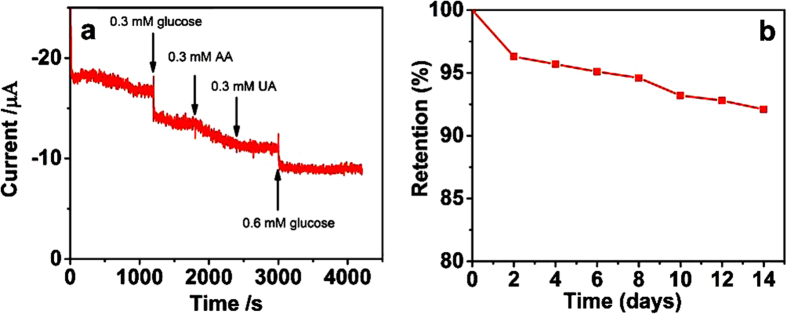
(**a**) Amperogram showing the effect of interfering compounds (0.3 mM of AA and 0.3 mM of UA) on the detection of glucose at a potential of −0.43 V. (**b**) The stability of the GOD-ZnO/CRG heterostructure on Pt electrode over a two-week storage period.

The GOD-ZnO/CRG/Pt electrode also exhibits excellent selectivity for glucose detection. As shown in Fig. 6a, the addition of interferents, e.g., 0.3 mM of ascorbic acid (AA) and 0.3 mM of uric acid (UA), in 0.1 M PBS containing 0.3 mM of glucose gives rise to small current changes, while a significant current response can be found for the subsequent addition of 0.6 mM of glucose. The response time is less than 3 s. To assess its stability, the GOD-ZnO/CRG/Pt electrode was stored at 4 °C after use and used to measure the current response for 0.3 mM of glucose every two days. The GOD-ZnO/CRG/Pt electrode retains 92% of its original current response over a storage period of 2 weeks (Fig. 6b), indicating a good shelf lifetime for the modified electrode.

## Conclusions

In summary, a heterostructure of ZnO and CRG has been obtained by hydrothermal growth of ZnO nanorods on CRG film, and was used as GOD immobilization electrode. Direct electrochemistry of GOD was achieved on heterostructure electrode. The properly aligned band structure of the ZnO/CRG heterostructure promoted the electron transfer from GOD-ZnO to electrode, and favored for the detection of the direct electrochemistry signal between GOD and ZnO-nanorods. Moreover, the heterostructure showed potential for glucose sensing. The study demonstrated for the first time that Pt can be used as electrode in ZnO based DET glucose biosensors and revealed that the band structure of electrode could influence the performance of direct electron transfer of GOD, and modulating the band structure would be helpful for the future design of electrodes in biosensors.

## Materials and Methods

### Synthesis and characterization of ZnO-nanorod/graphene heterostructure thin film

The heterostructure was prepared by hydrothermal growth of ZnO nanorods on CRG film. CRG was synthesized according to previously reported method^22^. The thin film of CRG was obtained by filtering method, which was then transferred onto a SiO_2_ substrate. Then, zinc acetate was spray-coated onto the CRG film by using an air brush with 10 mM zinc acetate/ethanol solution, with the substrate temperature at about 80 °C. Finally, ZnO nanorods were grown on the film in a solution containing equimolar concentrations (30 mM) of zinc nitrate hexahydrate (Zn(NO_3_)_2_ · 6H_2_O) and hexamethylenetetramine (C_6_H_12_N_4_, HMTA) at 90 °C for 1 h. The ZnO-nanorods/graphene heterostructure was characterized by scanning electron microscopy (SEM, JSM-7000F), transmission electron microscopy (TEM, JEM-2100), X-ray diffraction (XRD, XRD-6000, Cu Kα radiation) and photoluminescence spectra (PL, HR800, 325 nm). Fourier transform infrared spectroscopy (FT-IR) were recorded on a NEXUS870 spectrophotometer (USA).

### Biosensor device and electrochemical testing

To fabricate the glucose biosensor, the ZnO-nanorods/graphene film was transferred onto a standard Pt electrode with a diameter of 5 mm. ZnO nanorods directly grown on Pt electrode and graphene thin film on Pt electrode were taken as the controls. GOD solution was prepared by dissolving 40 mg GOD (~140 U mg^−1^) in 1 ml 0.02 M phosphate buffer solution (PBS). 20 μl GOD solution was dropped onto the electrode surface for immobilization, and dried under ambient condition. Then, 10 μl 5% (w/w) Nafion solution was dropped onto it and dried overnight at room temperature. All the electrochemical experiments were conducted on electrochemical workstation (CHI 660C), using conventional three-electrode electrochemical system with saturated calomel electrode (SCE) and platinum wire as the reference and counter electrodes, respectively.

## Additional Information

**How to cite this article**: Zhao, Y. *et al.* ZnO-nanorods/graphene heterostructure: a direct electron transfer glucose biosensor. *Sci. Rep.* **6**, 32327; doi: 10.1038/srep32327 (2016).

## Supplementary Material

Supplementary Information
