# Supplementary material for: ZnO-nanorods/graphene heterostructure: a direct electron transfer glucose biosensor
Source: Sci Rep. 2016 Aug 30;6:32327. doi: 10.1038/srep32327 (PMC5004169; doi:10.1038/srep32327)
Supplement: Supplementary Information [file srep32327-s1.doc]

**ZnO nanorods/graphene heterostructure: a direct electron transfer glucose biosensor**

Yu Zhao^a,1^, Wenbo Li^a,1^, Lijia Pan ^a,*^, Dongyuan Zhai^a^, Yu Wang^b^, Lanlan Li^a^, Wen Cheng^a^, Wei Yin^a^, Xinran Wang^a^, Jian-Bin Xu^c, a *^, Yi Shi^a, *^

^a^ School of Electronic Science and Engineering, Collaborative Innovation Center of

Advanced Microstructures, Nanjing University, Nanjing 210093, China

^b^ School of Chemistry and Chemical Engineering, Nanjing University, Nanjing 210093, China

^c^ Department of Electronic Engineering, The Chinese University of Hong Kong, Shatin, New Territories, Hong Kong SAR, China

*Correspondence Authors. Email: ljpan@nju.edu.cn (L. Pan), jbxu@ee.cuhk.edu.hk (J. B. Xu), and yshi@nju.edu.cn (Y. Shi)

^1^ These authors contributed equally to this work.


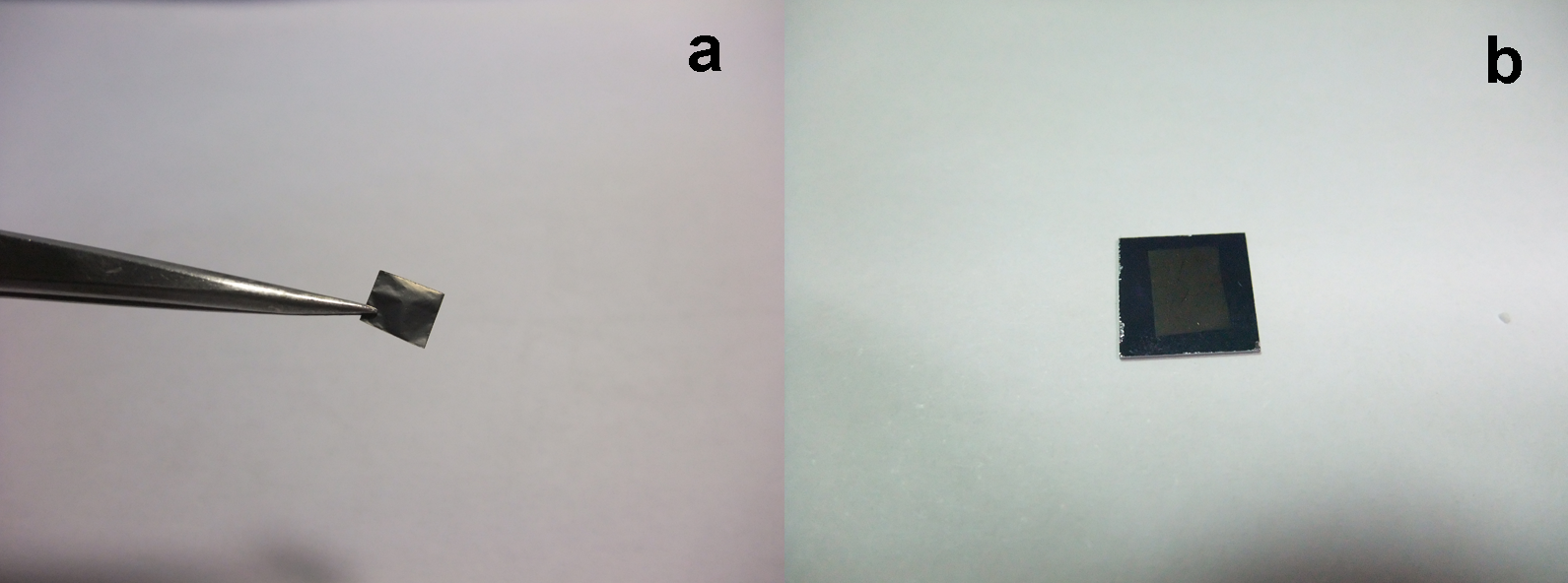


**Figure S1.** CRG film **(a)** without substrate and **(b)** on SiO_2_ substrate.


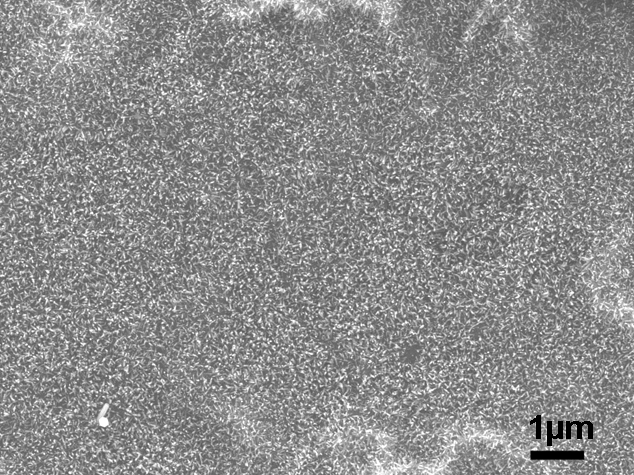


**Figure S2.** Low magnification SEM image of ZnO/CRG heterojunction


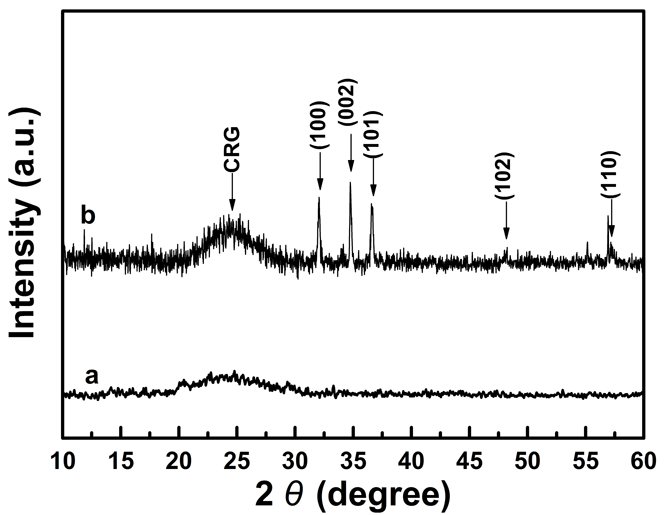


**Figure S3.** XRD patterns of **(a)** CRG film and (b) ZnO/CRG heterojunction


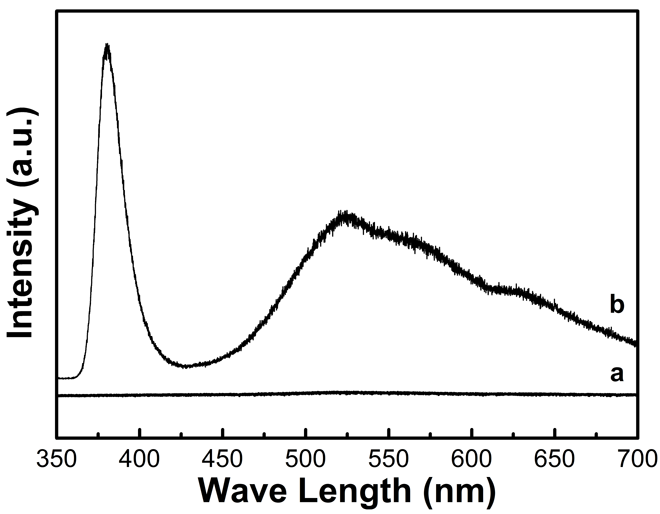


**Figure S4.** Photoluminescence spectra of **(a)** CRG film and **(b)** ZnO/CRG film

**Table S1** Comparison of the direct electrochemical glucose biosensors

| **Electrode** | **Linear range (mM)** | **Sensitivity**  **(μA mM^-1^ cm^-2^)** | | **Response**  **time (s)** | **Ref.** |
| --- | --- | --- | --- | --- | --- |
| ZnO/CRG/Pt | 0.2-1.6 | 89.84 | 3 | | this work |
| rGO–ZnO/GC | 0.2-6.6 | 13.7 | 4 | | [[1]](#endnote-2) |
| Chitosan/Vulcan carbon | 1-40 | 13 | 10 | | [[2]](#endnote-3) |
| Carbon–ZnO nanowire/GC | 0.01-1.6 | 35.3 | 5 | | [[3]](#endnote-4) |
| CdTe-CNTs/GC | <0.7 | 14.57 | - | | [[4]](#endnote-5) |
| BCNTs/GC | 0.05-0.3 | 111.57 | - | | [[5]](#endnote-6) |
| Graphene–chitosan/GC | 0.08-12 | 37.93 | - | | [[6]](#endnote-7) |
| Chitosan-laponite/Au | 0.01-5 | 33.9 | 5 | | [[7]](#endnote-8) |

**References**

1. 1 Dey, R. S. & Raj, C. R. Redox-functionalized graphene oxide architecture for the development of amperometric biosensing platform. *ACS Appl. Mater. Interfaces* 5, 4791–4798 (2013). [↑](#endnote-ref-2)
2. Mutyala, S. & Mathiyarasu, J. Direct electron transfer at a glucose oxidase – chitosan-modified vulcan carbon paste electrode for electrochemical biosensing of glucose. *Appl. Biochem. Biotechnol.* 172**,** 1517–1529 (2014). [↑](#endnote-ref-3)
3. Liu, J. *et al.* Electrochemistry communications carbon-decorated ZnO nanowire array : a novel platform for direct electrochemistry of enzymes and biosensing applications. *Electrochem. Commun.* **11,** 202–205 (2009). [↑](#endnote-ref-4)
4. Liu, Q., Lu, X., Li, J., Yao, X. & Li, J. Direct electrochemistry of glucose oxidase and electrochemical biosensing of glucose on quantum dots/carbon nanotubes electrodes. *Biosens. Bioelectron*. 22, 3203–3209 (2007). [↑](#endnote-ref-5)
5. Deng, C. et al. Direct electrochemistry of glucose oxidase and biosensing for glucose based on boron-doped carbon nanotubes modified electrode. *Biosens. Bioelectron*. 23, 1272–1277 (2008). [↑](#endnote-ref-6)
6. Kang, X. et al. Glucose Oxidase-graphene-chitosan modified electrode for direct electrochemistry and glucose sensing. *Biosens. Bioelectron*. 25, 901–905 (2009). [↑](#endnote-ref-7)
7. Shi, Q., Li, Q., Shan, D., Fan, Q. & Xue, H. Biopolymer-clay nanoparticles composite system (Chitosan-laponite) for electrochemical sensing based on glucose oxidase. *Mater. Sci. Eng. C* 28, 1372–1375 (2008). [↑](#endnote-ref-8)
